# Tetraiododiborane(4) (B_2_I_4_) is a Polymer Based on sp^3^ Boron in the Solid State

**DOI:** 10.1002/anie.201913590

**Published:** 2020-01-30

**Authors:** Jonas H. Muessig, Polina Lisinetskaya, Rian D. Dewhurst, Rüdiger Bertermann, Melanie Thaler, Roland Mitrić, Holger Braunschweig

**Affiliations:** ^1^ Institute for Inorganic Chemistry Julius-Maximilians-Universität Würzburg Am Hubland 97074 Würzburg Germany; ^2^ Institute for Sustainable Chemistry & Catalysis with Boron Julius-Maximilians-Universität Würzburg Am Hubland 97074 Würzburg Germany; ^3^ Institute for Physical and Theoretical Chemistry Julius-Maximilians-Universität Würzburg Am Hubland 97074 Würzburg Germany

**Keywords:** boron, density functional theory, diborane, halides, solid-state structure

## Abstract

Herein we present the first solid‐state structures of tetraiododiborane(4) (B_2_I_4_), which was long believed to exist in all phases as discrete molecules with planar, tricoordinate boron atoms, like the lighter tetrahalodiboranes(4) B_2_F_4_, B_2_Cl_4_, and B_2_Br_4_. Single‐crystal X‐ray diffraction, solid‐state NMR, and IR measurements indicate that B_2_I_4_ in fact exists as two different polymeric forms in the solid state, both of which feature boron atoms in tetrahedral environments. DFT calculations are used to simulate the IR spectra of the solution and solid‐state structures, and these are compared with the experimental spectra.

## Graphical abstract

**Tales of the unexpected**: The first solid‐state structures of tetraiododiborane(4) (B_2_I_4_) are presented, a compound that was long believed to exist as discrete molecules in all phases like the lighter tetrahalodiboranes(4) B_2_F_4_, B_2_Cl_4_, and B_2_Br_4_. In fact, B_2_I_4_ exists as two different polymeric forms in the solid state.

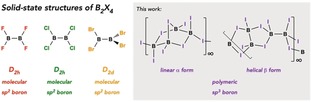

Ever since Stock's low‐yielding synthesis of B_2_Cl_4_ in 1925,^1^ the structures of the tetrahalodiboranes(4) (B_2_X_4_; X=F, Cl, Br, I) in their various phases (solid, liquid, gas) have been of fundamental interest to synthetic chemists.^2^, ^3^ The structures of tetrahalodiboranes(4) were revisited around the turn of the millenium by theoretical groups, thanks to more reliable modern computational methods.^4^ However, despite the ubiquity of diborane(4) species as reagents in organic chemistry,^5^ experimental studies of tetrahalodiboranes(4) remain very rare, and have long been hampered by their difficult syntheses and/or instability.

The structures of Lewis acidic Group 13 compounds are often influenced by the vacant p orbitals of the Group 13 atoms themselves, which can be stabilized in a number of different ways. Interactions of the empty p orbital of Group 13 “anes” (EX_3_; E=Group 13 element) with nearby electron lone pairs form the basis of a number of polymeric species,^6^ and are common methods of stabilization adopted by the higher homologues of boranes (e.g. AlBr_3_ and AlI_3_). In contrast, corresponding bridging halide interactions are negligible for most haloboranes (e.g. BX_3_ and B_2_X_4_; X=F, Cl, Br),^7^ except under high pressures.^8^ The interactions which are known to have the most influence on the conformations of tetrahalodiboranes(4) are hyperconjugation and π‐donation from the halide substituents. B_2_F_4_ was found to have a small rotational barrier between the planar (*D*_2*h*_) and the perpendicular (*D*_2*d*_) geometries;^4^ however, X‐ray and electron diffraction techniques, as well as Raman and IR spectroscopy, have verified tetrafluorodiborane(4) that adopts the planar (*D*_2*h*_) geometry in all phases (Figure 1).2a, 4c, 9 B_2_Cl_4_ is known to be perpendicular (*D*_2*d*_) in the gas and liquid phases,2c, 2e, 9d, 10 but to be planar (*D*_2*h*_) in the solid phase.2b, 9d, 10, 11 The heavier and sterically more demanding B_2_Br_4_ results in the perpendicular (*D*_2*d*_) form in all phases.2f, 2i

**Figure 1:**
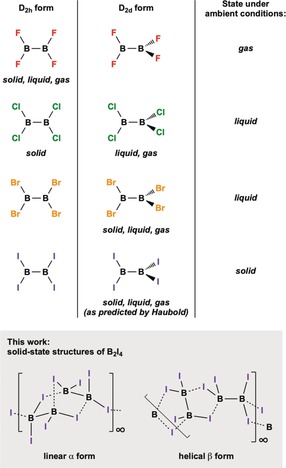
Geometries of the tetrahalodiboranes(4) in their various physical states (top) and the infinite polymeric solid‐state forms of B_2_I_4_ presented herein (bottom). Note that dotted B–I interactions are used to highlight each B_2_I_4_ unit for clarity, and do not imply weaker bonds or longer B–I distances.

The heaviest tetrahalodiborane(4), B_2_I_4_, is perhaps the least well‐understood of this particularly underexplored family of compounds. Haubold predicted B_2_I_4_ to be perpendicular in all phases, based on IR spectra of solid material as a nujol mull and on assumption of structural similarity to B_2_Br_4_ (Figure 1).^12^ It should be noted that the IR spectra also showed contamination with boron triiodide and further decomposition products which may have arisen from the nujol trituration process. To our knowledge, the only other spectroscopic data reported for B_2_I_4_ is its solution ^11^B NMR resonance (*δ*=70 ppm).^12^, ^13^

In recent years, interest in the reactivity of tetrahalodiboranes(4) has almost exclusively come from our laboratories and from the group of Kinjo, where B_2_Br_4_ has been used for the construction of molecules with boron–boron multiple bonds such as diborenes and diborynes,^3^, ^14^ as well as precursors to bis(boratabenzene) ligands.^15^ We have recently broadened our interests in tetrahalodiboranes(4), developing simple solution‐phase techniques to prepare the remaining B_2_X_4_ species (i.e. X=F, Cl, I) from B_2_Br_4_.^13^ Our subsequent explorations of the reactivity of B_2_I_4_ have demonstrated that this reactive molecule differs significantly from the lighter tetrahalodiboranes(4) in terms of its reactivity with low‐valent metal complexes,^16^ and that while (like B_2_Br_4_) it can be used as a precursor for the synthesis of dihalodiborenes, the products showed dramatic differences from their bromo analogues.^17^ B_2_I_4_ has also since been subjected to twofold quaternization of its boron atoms in the form of double addition of Lewis bases (providing adducts of the form B_2_I_4_L_2_)^18^ and halide anions (providing the hexaiododiborate dianion, [B_2_I_6_]^2−^).^19^

Despite the high synthetic potential of B_2_I_4_ in main‐group, transition‐metal, organic, and materials chemistry, there remains almost no experimental evidence for its structure, and no solid‐state structures have been reported. Herein we report two solid‐state structures of B_2_I_4_, showing the compound to have (at least) two crystal forms, both of which are polymers based on intermolecular B–I interactions. These polymeric structures are drastically different from those of the lighter tetrahalodiboranes(4), which exist as discrete molecules with planar, sp^2^‐hybridized boron atoms in the solid state. These findings are supported by IR and solid‐state NMR spectroscopy and computational studies.

Treatment of B_2_Br_4_ or B_2_Cl_4_ with 1.33 equiv of BI_3_ leads to the formation of solid B_2_I_4_, which can be easily isolated by removal of the BCl_3_ or BBr_3_ byproducts by vacuum distillation.^13^ Sublimation at 1×10^−3^ mbar/45 °C yielded colorless crystals of tetraiododiborane(4), which were used for the spectroscopic studies presented herein. It should be noted that the amorphous material obtained prior to sublimation provided effectively identical NMR and IR data. The IR spectrum of B_2_I_4_ in toluene solution matches that reported by Haubold, which is consistent with a perpendicular *D*_2*d*_ conformation (Figure 2). However, the IR spectrum of the solid material recrystallized from toluene at −30 °C did not show the typical bands expected for either the *D*_2*d*_ or *D*_2*h*_ forms of the molecule (vide infra), thereby indicating another structural motif.

**Figure 2:**
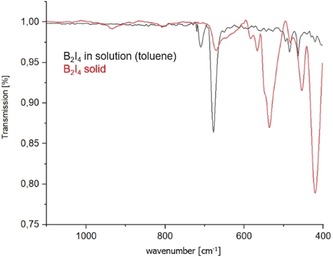
Solution and solid‐state IR spectra of B_2_I_4_.

In order to gain a better picture of the solid‐state structure of B_2_I_4_, ^11^B RSHE/MAS (RSHE=rotor‐synchronized Hahn‐Echo, MAS=magic angle spinning) NMR spectra were recorded for the amorphous and crystalline material. In both spectra a broad resonance at *δ*=−16 ppm (FWHH=2940 Hz) was detected, which is far upfield from the corresponding resonance observed in the solution ^11^B NMR spectrum (*δ*=70 ppm) and suggests that the boron centers in the compound are quaternized in the solid state. Comparison of the chemical shift of this resonance with the solution ^11^B NMR resonances of Lewis base adducts [B_2_I_4_(SMe_2_)_2_] (*δ*=−20 ppm),^13^ [B_2_I_4_(PMe_3_)_2_] (*δ*=−27 ppm),^17^ and [B_2_I_4_(PCy_3_)_2_] (*δ*=−27 ppm)^17^ support this assumption. Additional sharp minor signals detected in the ^11^B RSHE/MAS NMR spectra can be assigned to liquid B_2_I_4_ (*δ*=70 ppm), liquid BI_3_ (*δ*=−5 ppm) and further liquid decomposition products (*δ*=56, 19 ppm) (see the Supporting Information). This mixture can be explained by partial melting of the sample caused by pressure arising from MAS conditions (rotation at 15 kHz), and presumably subsequent decomposition of B_2_I_4_. Experiments conducted with lower rotation rates (7–11 kHz) led to less decomposition of the sensitive compound, but did not eliminate the melting or the formation of the decomposition products.

Confirmation of the nonplanar boron atoms of B_2_I_4_ in the solid state was unequivocally provided by single‐crystal X‐ray diffraction of three separate samples. Single crystals grown from saturated toluene solutions at −30 °C provided one crystal form of the compound (α form; Figure 3, top), while crystallization at ambient temperature or by sublimation (40–45 °C, <1×10^−3^ mbar) provided a second form (β form; Figure 3, bottom). In both isomers the B_2_I_4_ units are connected via bridging iodides, which bridge boron atoms in a symmetrical fashion such that the two B–I distances of each bridging iodide are effectively identical, resulting in infinite 1D polymers. In the α form, dimers of B_2_I_4_ units form through three bridging iodides, leading to a norbornane‐like [2.2.1] bicyclic structure formed from two five‐membered B_3_I_2_ rings. Each dimer is connected to two other dimers in a linear fashion through two further B–I bonds. The β form is also made up of B_2_I_4_ dimers. In each dimer, two B_2_I_4_ units are connected through two fused monocyclic B_3_I_2_ rings, forming an edge‐shared [3.3.0] bicyclic structure. On the supramolecular level, the α form adopts a roughly linear structure, while the β form has a distinct helical superstructure with a pitch of ca. 10.7 Å.

**Figure 3:**
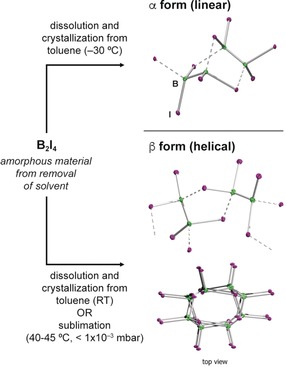
Linear (α) and helical (β) solid‐state forms of B_2_I_4_ as derived from single‐crystal X‐ray diffraction. Note that dotted B–I interactions are used to highlight each B_2_I_4_ unit for clarity, and do not imply weaker bonds or longer B–I distances.

The B−B bond lengths of the two solid‐state forms of B_2_I_4_ are almost identical (α form: 1.695(6) Å; β form: 1.68(2) Å), although the high experimental error in the latter precludes more detailed analysis. As expected, the B–I distances (2.301(4)–2.336(4) Å) involving bridging iodides are significantly longer than those of terminal iodides (α form: 2.184(4)–2.193(4) Å; β form: 2.189(9)–2.180(9) Å).

Theoretical simulations were performed to establish a correlation between the structural and spectral properties of B_2_I_4_ in solution and in its two solid‐state forms. Firstly, geometry optimization confirmed that the minimum‐energy structure of molecular B_2_I_4_ in toluene solution possesses *D*_2*d*_ symmetry. Secondly, the calculated IR spectrum of an isolated B_2_I_4_ molecule in the *D*_2*d*_ conformation is in very good agreement with the experimental solution IR spectrum (Figure 4) and with the assignment of vibrations made by Haubold,^12^ confirming the *D*_2*d*_‐symmetric geometry of B_2_I_4_ in solution. The theoretical IR spectrum represents a weighted sum of vibrational spectra of B_2_I_4_ species with different isotopic compositions corresponding to a relative abundance of ^10^B and ^11^B isotopes in a ratio of 20:80 (see the Supporting Information for individual spectra of the isotopomers).

**Figure 4:**
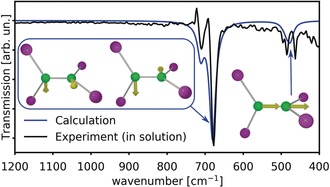
Comparison of the experimental solution‐state (toluene) IR spectrum of B_2_I_4_ with the simulated IR spectrum of isolated B_2_I_4_ molecules (*D*_2*d*_ symmetry). The most intense vibrational modes are presented as insets.

Simulations of the vibrational spectra of both solid‐state structures (α and β forms) were also performed. The calculated spectra of the two crystal forms possess noticeably distinct features, as shown in Figure 5 a (green line) and 5b (blue line). The three most intense vibrational modes of the helical β structure represent deformation (A), rotation (B), and shifting (C) of the boron skeleton, as shown in Figure 5 c (see Movies A, B, and C provided in the Supporting Information). The experimental solid‐state IR spectrum of B_2_I_4_ (Figure 5 a,b, black line) is poorly fit by the calculated IR of the α form (Figure 5 a), but is fit very well by that of the β form (Figure 5 b). Although the crystals used for the IR spectrum were grown at −30 °C from toluene, and thus should correspond to the α form, a change to the β form likely takes place when the sample is ground and pressed into a pellet for the measurement. This hypothesis is in line with the pressure sensitivity of B_2_I_4_ observed during the fast rotation of the sample when we measured the solid‐state MAS NMR spectra. It should also be noted that the amorphous material obtained from solvent evaporation (Figure 3) provides an essentially identical solid‐state IR spectrum. The moderately intense vibrational band at ca. 670 cm^−1^, which is completely absent in the simulated IR spectra, can be attributed to a minor fraction of molecular B_2_I_4_ in the *D*_2*d*_ form, as established in solutions of the compound.

**Figure 5:**
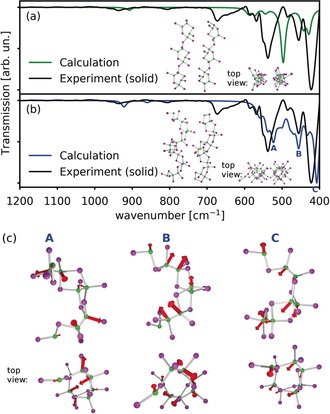
a,b) Comparison of the experimental (solid‐state) spectrum of B_2_I_4_ with the simulated IR spectra of the linear (a) and helical (b) forms of B_2_I_4_. c) Depiction of the vibration modes corresponding to bands A, B, and C in (b).

An unequivocal rationale for the differences in solid‐state structures between B_2_I_4_ and its lighter homologues is elusive, although the observed bridging of the iodide groups is indeed intuitive from consideration of the properties of the related monoboron halides BX_3_ (X=F to I). The Lewis acidity of BX_3_ species is generally acknowledged to increase in the order BF_3_<BCl_3_<BBr_3_<BI_3_, although the reasons behind this have been the subject of extensive debate.^20^ A theoretical study using the addition of two electrons to BX_3_ species (in order to model an extreme case of a Lewis base) found that the two‐electron affinity of the B atom of BX_3_ species increases as the halide is changed from F to I, while the energy required to pyramidalize the compounds also becomes lower.20e These two effects are likely also at play in the B_2_X_4_ series, overall making pyramidalization at boron and iodide bridging favorable in the solid‐state structure of B_2_I_4_ but not its lighter homologues.

In order to investigate the bonding in the solid‐state structures of B_2_I_4_(α) and B_2_I_4_(β) in more depth, an analysis based on the quantum theory of atoms in molecules (QTAIM)^21^ was performed. The QTAIM representation of the helical (β) structure is schematically shown in Figure 6. The electron density parameters at the bond critical points (BCPs) and the bonding analysis of the linear (α) structure are provided in the Supporting Information. The simulations reveal that, in both solid‐state structures, the strength of the iodide‐bridging bonds (B–I_B_, blue lines in Figure 6) is approximately 80 % of that of the terminal B–I bonds (B–I_T_, yellow lines), making them stable against cleavage. According to a reported theoretical study,^22^ the strengths of bridging halogen bonds relative to the terminal bonds increases from F to I, which makes the formation of halogen bridges possible for B_2_I_4_ but not for the lighter homologues. Moreover, our QTAIM calculations have uncovered the presence of BCPs between the iodides in the [B_2_I_4_]_*n*_ strands of both crystal forms, suggesting weak I⋅⋅⋅I interactions based on dispersion forces (magenta lines in Figure 6). These calculated intrastrand I⋅⋅⋅I interactions add a further stabilizing effect to a polymeric species that would not be possible with the lighter homologues B_2_X_4_ (X=F, Cl, Br).

**Figure 6:**
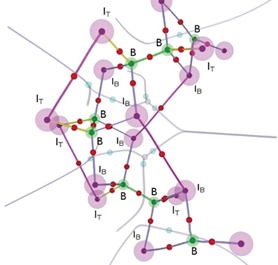
QTAIM representation of the solid‐state structure of B_2_I_4_(β). Semitransparent spheres denote the positions of boron (B), terminal iodide (I_T_), and bridging iodide (I_B_) atoms. The following electron density critical points are depicted: attractors (black), bond critical points (red), ring critical points (light blue), and local minima (gray).

Herein we use IR spectroscopy, single‐crystal X‐ray diffraction, and DFT calculations to confirm the solution structure of B_2_I_4_ and establish its polymeric nature in the solid state. Two polymeric crystal forms are structurally authenticated, one of which possesses a distinct helical structure. Through computational simulation of the IR spectra of the two solid forms, the experimental solid IR spectrum of the samples is shown to correspond to the form adopted at higher temperatures. The massive discrepancies between the solid‐state structures of B_2_I_4_ and the lighter homologues B_2_X_4_ (X=F‐Br) further underline the fundamental differences observed in the few available reactivity studies,^16^, ^17^, ^18^, ^19^ and hint at more surprises to come in the chemistry of the tetrahalodiboranes(4).

## Conflict of interest

The authors declare no conflict of interest.

## Supporting information

As a service to our authors and readers, this journal provides supporting information supplied by the authors. Such materials are peer reviewed and may be re‐organized for online delivery, but are not copy‐edited or typeset. Technical support issues arising from supporting information (other than missing files) should be addressed to the authors.

Supplementary

Supplementary

Supplementary

Supplementary
