# Supplementary material for: Tetraiododiborane(4) (B2I4) is a Polymer Based on sp3 Boron in the Solid State
Source: Angew Chem Int Ed Engl. 2020 Jan 30;59(14):5531–5. doi: 10.1002/anie.201913590 (PMC7154622; doi:10.1002/anie.201913590)
Supplement: Supplementary file 1 — Supplementary [file ANIE-59-5531-s001.pdf]

Angewandte Chemie
Eine Zeitschrift der Gesellschaft Deutscher Chemiker

Supporting Information

Tetraiododiborane(4) (B_2I_4) is a Polymer Based on sp^3 Boron in the Solid State

Jonas H. Muessig, Polina Lisinetskaya, Rian D. Dewhurst, Rüdiger Bertermann, Melanie Thaler, Roland Mitrić, and Holger Braunschweig**

anie_201913590_sm_miscellaneous_information.pdf
anie_201913590_sm_movie_A.avi
anie_201913590_sm_movie_B.avi
anie_201913590_sm_movie_C.avi

Synthetic Details

General considerations. All manipulations were performed under an inert atmosphere of dry argon using either standard Schlenk-line or glovebox techniques. Deuterated solvents were dried over molecular sieves and degassed by three freeze-pump-thaw cycles prior to use. All other solvents were distilled from appropriate drying agents.[1] Solvents were stored under argon over activated molecular sieves. NMR spectra were recorded on a Bruker Avance I 400 (^{1}H, 400.1 MHz; ^{11}B, 128.4). Chemical shifts are given in ppm and were referenced in solution to external $BF_3{\cdot}OEt_2$ (^{11}B). The solid-state ^{11}B RSHE/MAS (RSHE = rotor synchronized Hahn-Echo, MAS = magic angle spinning) NMR spectra of B_2I_4 were recorded using a Bruker DSX-400 spectrometer operating at 128.4 MHz (^{11}B) using a 4 mm (o. d.) ZrO_2 rotor at spinning frequencies between 7 and 15 kHz, 300 scans were accumulated. The ^{11}B NMR shifts in the solid state are given in ppm and were referenced externally by adjusting the field of the NMR spectrometer to set the low-field ^{13}C signal of adamantane to 38.48 ppm to comply with IUPAC recommendations for reference. BI_3 was purchased from commercial sources and used without further purification. B_2I_4[2] was prepared according to a literature procedure and sublimed at 40-45 ºC under vacuum ($< 1 \times 10^{-3}$ mbar). The synthesis of B_2I_4 was carried out in brown-glass Schlenk flasks to exclude light.

NMR spectra of B_2I_4:

^{11}B NMR (*d_8*-toluene):

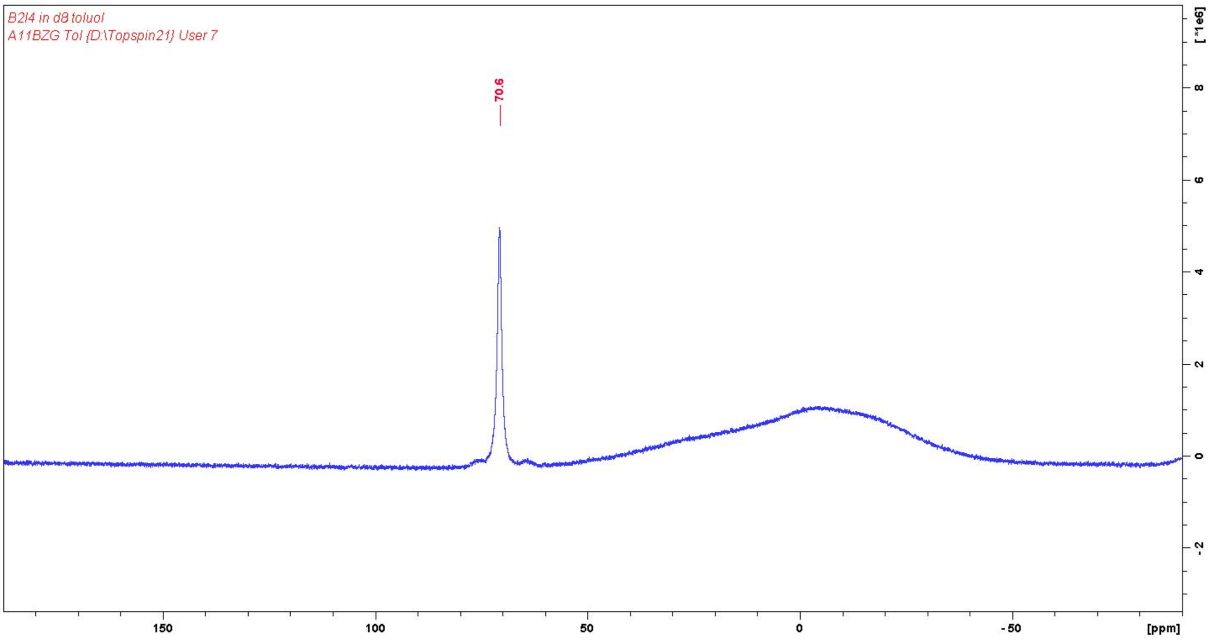

^{11}B NMR (*d_8*-toluene): *after decomposition at ambient temperature*

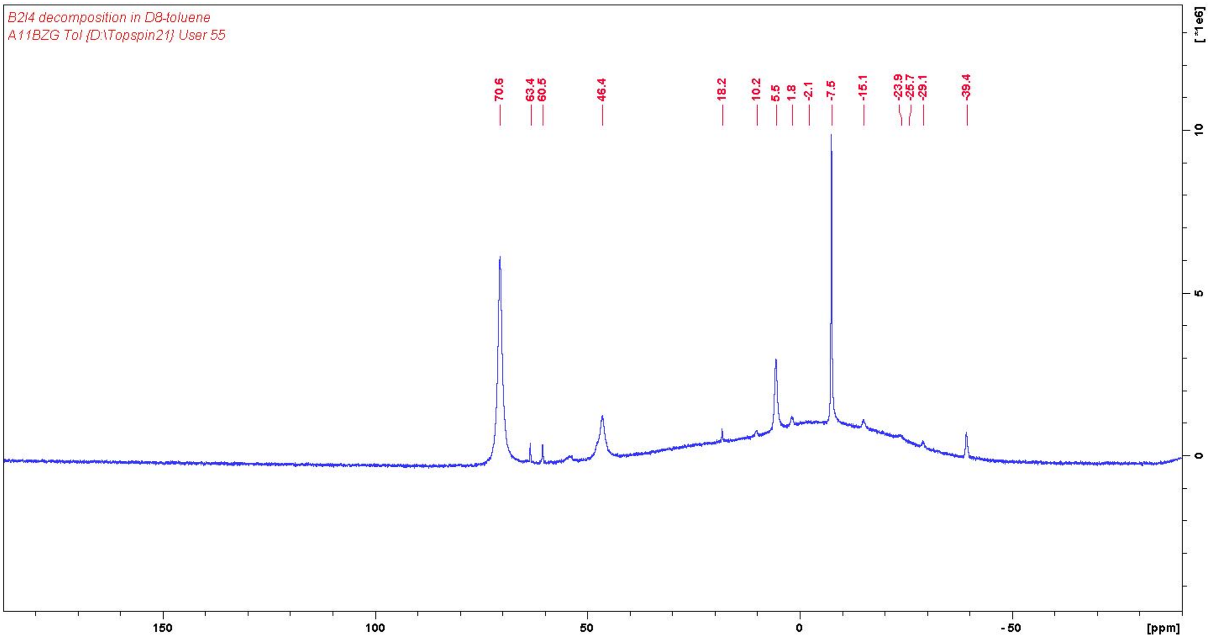

Solid-state ^{11}B RSHE/MAS at 15 kHz:

Note: sharp signals are assigned to liquid species, which are B_2I_4 and its decomposition products. Pressure caused by the rotation of the sample effects a partial change of the state from solid to liquid and decomposition of B_2I_4. The signals at 100 and 112 ppm are spinning side bands from the MAS at 15000 Hz arising from the signals at –4.7 and –16 ppm.

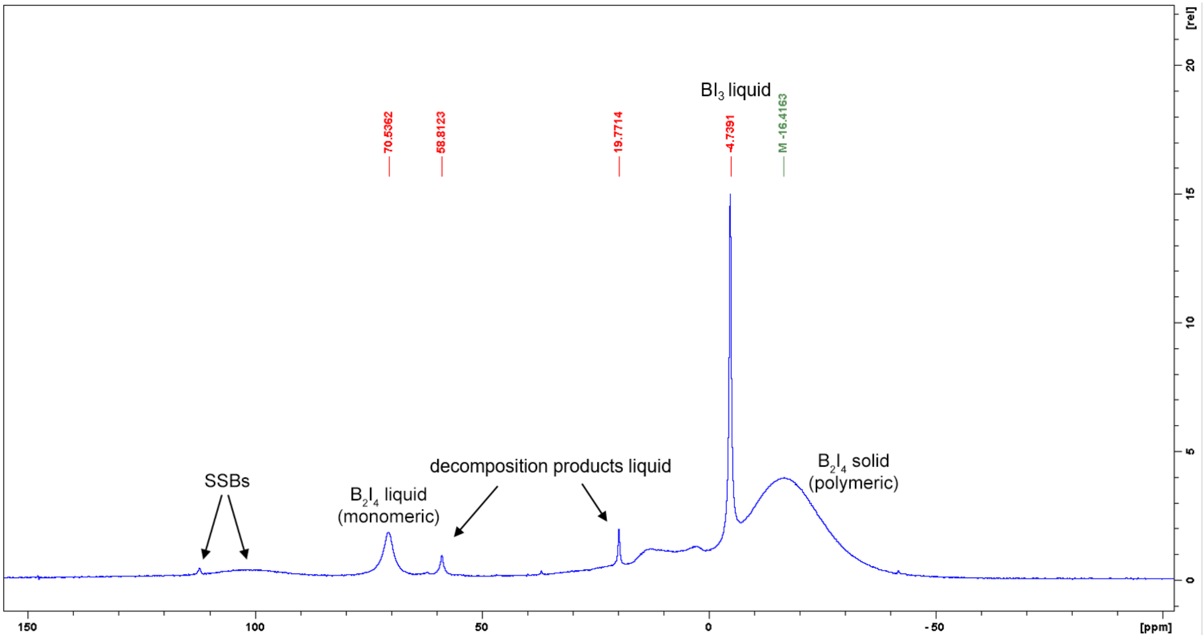

IR spectra of solid, neat B_2I_4 (upper spectrum) and in toluene solution (lower spectrum):

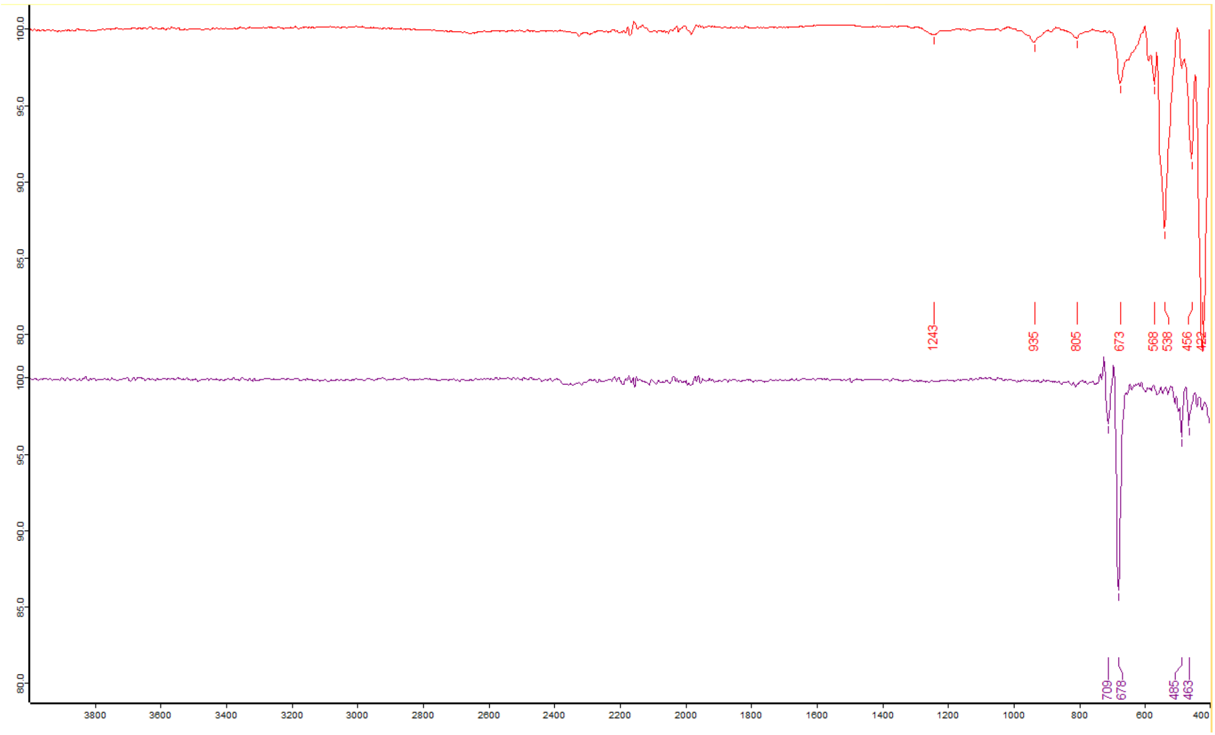

Crystallographic Details

The crystal data of $B_2I_4(\alpha)$ and $B_2I_4(\beta)$ were collected on a BRUKER D8 QUEST diffractometer with a CMOS area detector and multi-layer mirror monochromated $Mo_{K\alpha}$ radiation. The structure was solved using intrinsic phasing methods[3] refined with the SHELXL program[4] and expanded using Fourier techniques. All non-hydrogen atoms were refined anisotropically.

Crystal data for $B_2I_4(\alpha)$: B_2I_4, $M_r = 529.22$, colorless block, $0.13\times0.10\times0.03$ mm^3, monoclinic space group $P2/c$, $a = 7.545(2)$ Å, $b = 7.0128(14)$ Å, $c = 13.798(3)$ Å, $\beta = 96.691(16)°$, $V = 725.1(3)$ Å^3, $Z = 4$, $\rho_{calcd} = 4.848$ g·cm^{-3}, $\mu = 17.063$ mm^{-1}, $F(000) = 888$, $T = 100(2)$ K, $R_1 = 0.0222$, $wR^2 = 0.0370$, 1489 independent reflections [$2\theta \leq 52.742°$] and 57 parameters. CCDC-1955573.

Crystal data for $B_2I_4(\beta)$: The diffraction pattern of β-form showed smudging of reflections. Depending on integration settings, the distances B1–B1' and B2–B2' were found to be in the ranges of 1.677(0.018)–1.684(0.018) Å and 1.672(0.017)–1.688(0.017) Å respectively. We consider this data not precise enough to use it in discussion of geometric parameters. B_8I_{16}, $M_r = 2116.88$, yellow plate, $0.15\times0.05\times0.024$ mm^3, tetragonal space group $P4_12_12$, $a = 11.664(6)$ Å, $b = 11.664$ Å, $c = 10.682(6)$ Å, $\alpha = 90°$, $\beta = 90°$, $\gamma = 90°$, $V = 1453.1(18)$ Å^3, $Z = 2$, $\rho_{calcd} = 4.838$ g·cm^{-3}, $\mu = 17.030$ mm^{-1}, $F(000) = 1776$, $T = 103(2)$ K, $R_1 = 0.0217$, $wR^2 = 0.0453$, 1542 independent reflections [$2\theta \leq 53.468°$] and 55 parameters. The Uii displacement parameters of atoms B2 were restrained with the ISOR keyword to approximate isotropic behavior. CCDC-1955572.

Computational details

The IR spectrum of single B_2I_4 molecules possessing D_{2d} symmetry were simulated employing density-functional theory (DFT) with the gradient-corrected exchange-correlation functional of Perdew, Burke und Ernzerhof (PBE)[5] and the triple-zeta valence plus polarization (TZVP)[6] basis set of Ahlrichs. Prior to the spectral calculations the nuclear geometry of the B_2I_4 molecule was optimized at the same level of theory. The calculations were carried out using the Gaussian09 program package.[7]

The IR spectra calculations of the B_2I_4 (linear) and B_2I_4 (helical) structures were performed at the periodic plane wave DFT level of theory as implemented in the Quantum ESPRESSO 6.2 suite of programs.[8,9] The PBE functional was employed in the DFT simulations. The repulsive part of the Kohn-Sham potential was taken into account using the scalar-relativistic pseudopotentials, which were generated using the projector augmented wave (PAW)[10] method. The Kohn-Sham molecular orbitals of B_2I_4 were represented using the plane-wave basis set with energy cut-offs of 125 Ry and 175 Ry for the linear and helical structures, respectively. The corresponding charge density cut-offs were 1000 Ry and 1250 Ry. The first Brillouin zone was sampled using the equally spaced 4x4x4 and 3x3x3 Monkhorst-Pack k-point grids[11] for the linear and helical structures. The energy and electron density cut-offs as well as the size of the grid in the reciprocal space were chosen following the total energy convergence criterion.

In the simulations, the optimal geometries of the unit cell of both structures were first determined by performing the full atomic+cell relaxation. For the optimized unit cells the IR spectra were calculated at the Γ-point using the density-functional perturbation theory. The resulting spectra were broadened with Lorentzian shape functions with a full width at half maximum of 35 cm^{-1}.

The bonding analysis was performed employing the quantum theory of atoms in molecules (QTAIM).[12] The electron density, its critical points, and density properties were calculated using the DGrid program package.[13]

Nuclear geometry (B_2I_4 in the D_{2d} form, Å)

I	0.000000	1.857057	1.868043
I	0.000000	-1.857057	1.868043
I	1.857057	0.000000	-1.868043
I	-1.857057	0.000000	-1.868043
B	0.000000	0.000000	-0.828037
B	0.000000	0.000000	0.828037

Nuclear geometry (B_2I_4 in the linear α form, Å)

I	6.01178347	4.47721795	5.37641744
I	0.6237439	4.47721795	2.30786301
I	5.02471708	3.56023877	13.24766236
I	-0.36332249	3.56023877	10.17910793
I	-0.49353055	1.27396954	3.93562246
I	-1.48059694	6.76348718	11.80686738
I	4.76807273	0.97638059	7.14534054
I	1.86744935	0.97638059	0.5389452
I	3.78100634	7.06107613	15.01658017
I	0.88038296	7.06107613	8.41019012
I	3.31776104	8.11789646	3.84214287
I	2.33069465	-0.08043974	11.7133825
I	4.36115006	3.17402645	2.22692601
I	2.27437731	3.17402645	5.45735973
I	3.37408367	4.86343027	10.09816564
I	1.28731092	4.86343027	13.32860465
B	5.32366556	2.67244981	4.30910175
B	1.31185652	2.67244981	3.37518399
B	4.33659917	5.3650122	12.18034138
B	0.32479013	5.3650122	11.24642891
B	4.16536187	1.78151859	5.19250001
B	2.47016021	1.78151859	2.49178044
B	3.17830077	6.25593813	13.06374493
B	1.48309382	6.25593813	10.36302536

Nuclear geometry (B_2I_4 in the helical β form, Å)

I	5.33835589	8.16253348	5.67712749
I	7.53728722	4.71310963	0.10012912
I	4.71310963	7.53728722	11.05386762
I	1.09946302	1.72470928	8.26537108
I	11.7761748	11.15092854	2.68837271
I	8.16253348	5.33835589	5.47686925
I	11.15092854	11.7761748	8.46562403
I	1.72470928	1.09946302	2.88862566
I	7.89581168	7.81407589	8.25500797
I	4.97982614	5.06156193	2.6780096
I	5.06156193	4.97982614	8.47599243
I	11.41765034	1.37625698	5.6874906
I	1.45799277	11.49938084	0.11049223
I	7.81407589	7.89581168	2.89899406
I	11.49938084	1.45799277	11.04350451
I	1.37625698	11.41765034	5.46650614
I	10.76145229	7.92380107	5.98860269
I	2.11419082	4.95184204	0.41160432
I	4.95184204	2.11419082	10.74239242
I	8.55200973	1.48597687	7.95389588
I	4.32362809	11.38966095	2.37689751
I	7.92380107	10.76145229	5.16539405
I	11.38966095	4.32362809	8.77709923
I	1.48597687	8.55200973	3.20010086
I	5.34330204	10.43608026	8.66943186
I	7.53234107	2.43955756	3.09243349
I	2.43955756	7.53234107	8.06156854
I	1.09452216	3.99826135	5.27306671
I	11.78112095	8.87737647	10.85006508
I	10.43608026	5.34330204	2.48457017
I	8.87737647	11.78112095	0.30393166
I	3.99826135	1.09452216	5.88093003
B	8.58248013	7.57919989	6.04965458

B	4.29316298	5.29644322	0.47265621
B	5.29644322	4.29316298	10.68134053
B	10.73098189	1.14137569	7.8928387
B	2.14466122	11.73426213	2.31584033
B	7.57919989	8.58248013	5.10434216
B	11.73426213	2.14466122	8.83815641
B	1.14137569	10.73098189	3.26115804
B	5.61438809	8.34277965	7.98658808
B	7.26124973	4.53286346	2.40958971
B	4.53286346	7.26124973	8.74440703
B	0.82343082	1.90495545	5.95591049
B	12.052207	10.97068237	0.37891212
B	8.34277965	5.61438809	3.16740866
B	10.97068237	12.052207	10.77508991
B	1.90495545	0.82343082	5.19809154

Simulated IR spectra of molecular B_2I_4 in D_{2d} form

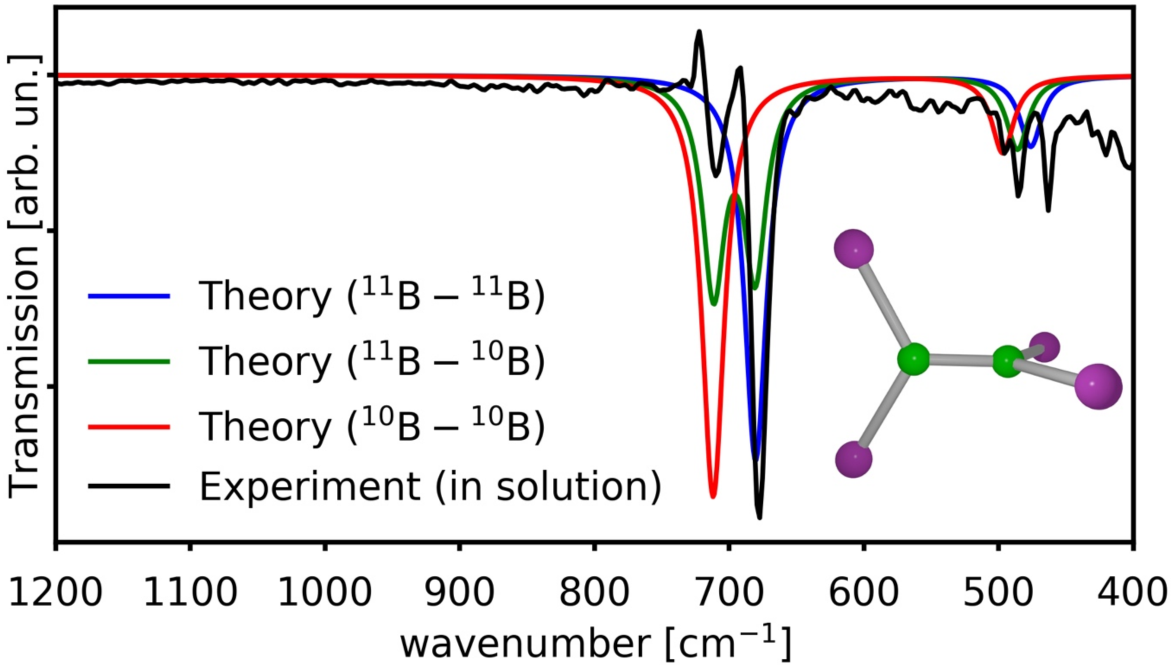

Three possible isotopic compositions of the B_2I_4 molecule are presented. Blue line: both boron atoms are common ^{11}B; green line: one boron atom is substituted with a ^{10}B isotope; red line: both boron atoms are substituted with ^{10}B isotopes.

Most intense normal modes of the linear α structure

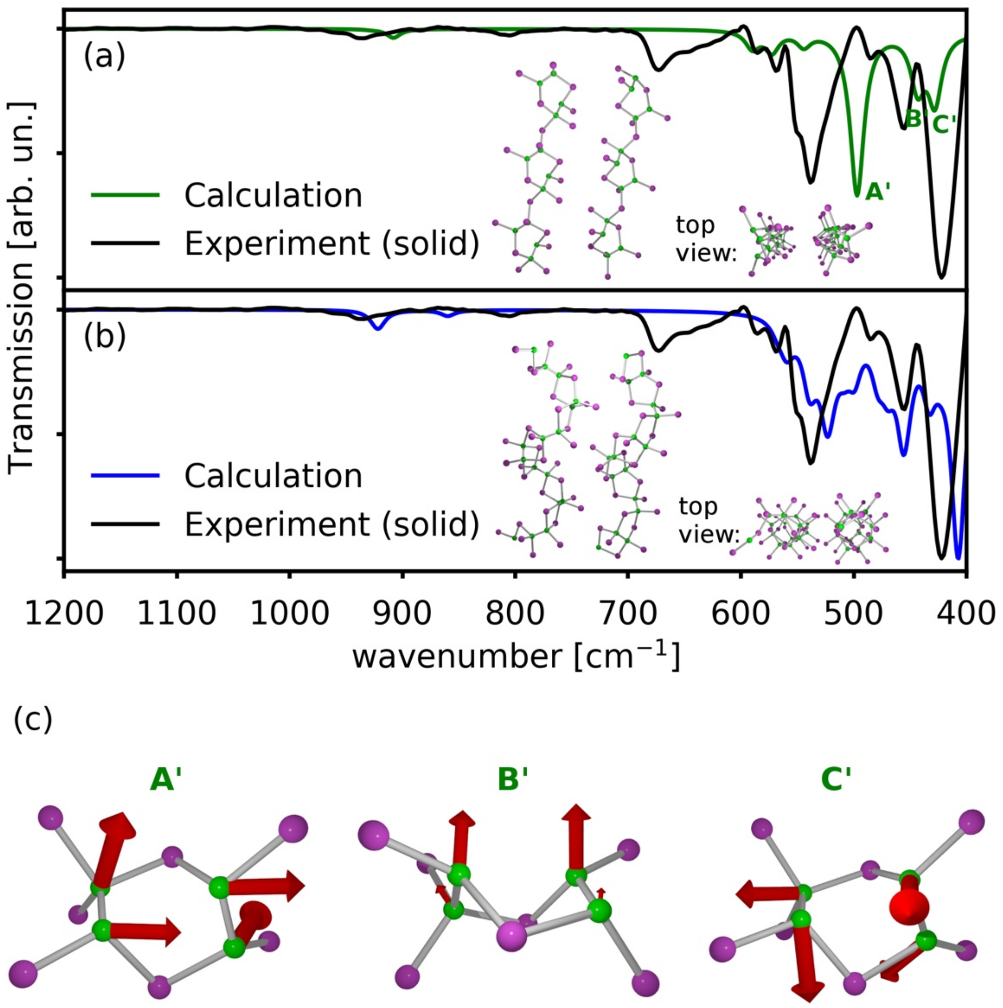

Bonding analysis

B_2I_4 in the linear α form

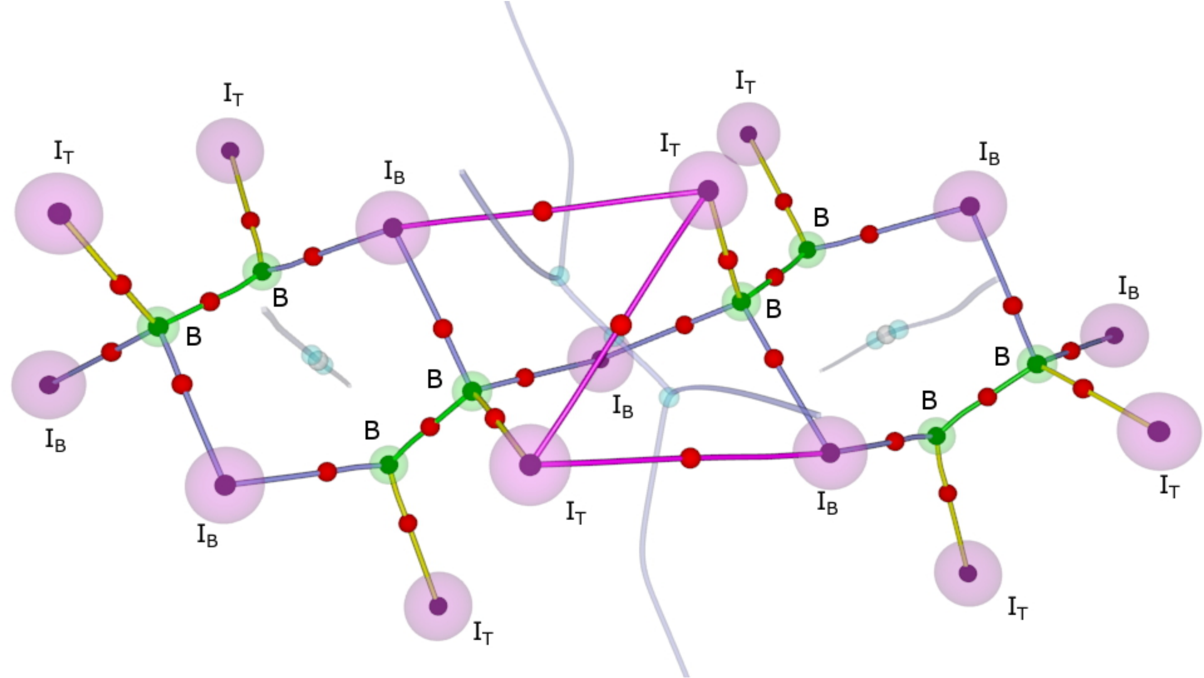

QTAIM representation of B_2I_4 in the linear α form (two segments of the linear chain). Semi-transparent circles denote positions of boron (green) and iodine (magenta) atoms. The following critical points are marked with small spheres: attractors (black), bond critical points (red), ring critical points (blue), local minima (gray). Solid lines represent the atomic interaction lines, corresponding to (green) B-B, (yellow) B-I_T, (blue) B-I_B, (magenta) I-I bonds.

In the structure two types of I atoms are present, bridging (I_B) and terminal (I_T). The bonding information is summarized in the table below. The following parameters are calculated at the bond critical points (BCP): electron density (ρ), its laplacian (Δρ), energy density (H). These parameters characterize a bond between atoms: ρ describes the strength of the bond, $\Delta\rho < 0$ means "shared interaction" (covalent or polar), $\Delta\rho > 0$ – "closed-shell interaction" (ionic, van der Waals, etc.), $H < 0$ stands for strong covalent and $H > 0$ for dispersive interactions.[14]

BCP	ρ	Δρ	H
B-B	0.149	-0.316	-0.1
B-I_T	0.101	-0.103	-0.05
B-I_B	0.078	-0.058	-0.032
I_T-I_T	0.0093	0.022	0.0007
I_T-I_B	0.0066	0.016	0.0007

The strengths of the B-I_B bonds are comparable to those of B-I_T bonds, which makes them stable against cleavage and supports formation of the B-I-B bridges. Additional stability of the polymer chain is provided by weak dispersive I…I interactions between monomers.

B_2I_4 in the helical β form

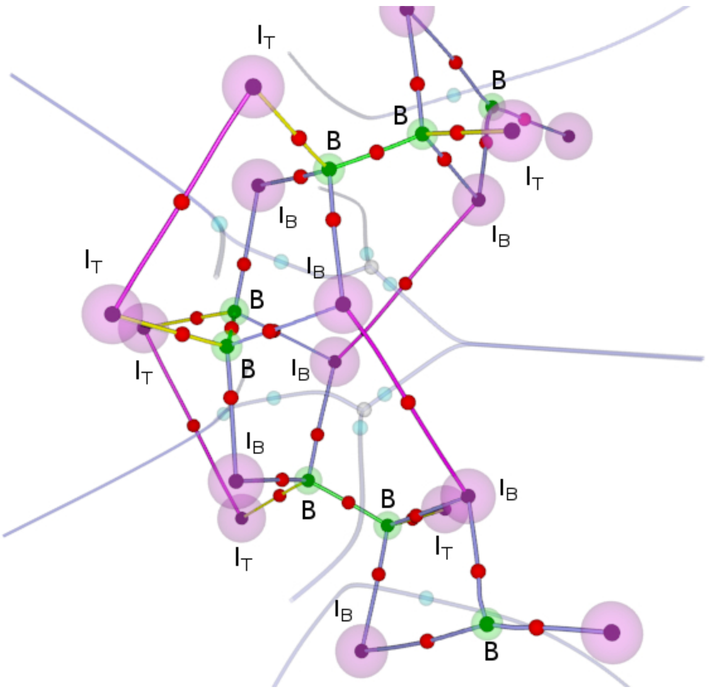

QTAIM representation of B_2I_4 in the helical β form (single helix turn). The notation is the same as for the linear structure.

In the helical β structure the bridging (I_B) and the terminal (I_T) iodide atoms are present as well. The bonding information is summarized in the table below.

BCP	ρ	Δρ	H
B-B	0.157	-0.348	-0.107
B-I_T	0.101	-0.115	-0.054
B-I_B	0.082	-0.069	-0.037
I_T-I_T	0.0061	0.014	0.0007
I_B-I_B	0.0064	0.016	0.0007

In this structure, the B-I_B bonds are also of comparable strength to the B-I_T bonds, which makes it possible to form B-I-B bridges like in the linear α structure. Weak dispersive I...I interactions not only provide additional bonding between individual monomers, but also stabilize the helical structure within a single monomer.

References

[1] D. D. Perrin, W. L. F. Armarego, *Purification of Laboratory Chemicals,* Pergamon Press, Oxford, 3rd ed., 1988.

[2] M. Arrowsmith, J. Böhnke, H. Braunschweig, A. Deißenberger, R. D. Dewhurst, W. C. Ewing, C. Hörl, J. Mies, J. H. Muessig, *Chem. Commun.* **2017**, *53*, 8265–8267.

[3] G. Sheldrick, *Acta Cryst.* **2015**, *A71*, 3–8.

[4] G. Sheldrick, *Acta Cryst.* **2008**, *A64*, 112–122.

[5] J. P. Perdew, K. Burke, M. Ernzerhof, *Phys. Rev. Lett.* **1996**, *77*, 3865–3868.

[6] F. Weigend, R. Ahlrichs, *Phys. Chem. Chem. Phys.* **2005**, *7*, 3297–3305.

[7] M. J. Frisch, G. W. Trucks, H. B. Schlegel *et al.*, Gaussian 09, Revision D.01, Gaussian Inc., Wallingford CT, 2016.

[8] P. Giannozzi *et al., J. Phys.: Condens. Matter* **2017**, *29*, 465901.

[9] www.quantum-espresso.org

[10] G. Kresse, D. Joubert, *Phys. Rev. B* **1999**, *59*, 1758–1775.

[11] H. J. Monkhorst, J. D. Pack, *Phys. Rev. B* **1976**, *13*, 5188–5192.

[12] R. Bader, *Atoms in Molecules: A Quantum Theory*, USA: Oxford University Press, 1994.

[13] M. Kohout, DGrid, version 4.6, Radebeul, 2011.

[14] R. Bader, *Chem. Rev.* **1991**, *91*, 893-928.
